# Relationship between sensory processing skills and motor skills in 12‐month‐old infants

**DOI:** 10.1002/brb3.70052

**Published:** 2024-09-24

**Authors:** Ramazan Yildiz, Ayse Yildiz, Rabia Zorlular, Bulent Elbasan

**Affiliations:** ^1^ Department of Physical Therapy and Rehabilitation, Faculty of Health Sciences Erzurum Technical University Erzurum Turkey; ^2^ Department of Physical Therapy and Rehabilitation, Bor Faculty of Health Sciences Ömer Halisdemir University Nigde Turkey; ^3^ Department of Physical Therapy and Rehabilitation, Faculty of Health Sciences Gazi University Ankara Turkey

**Keywords:** child development, developmental outcomes, preterm infants, sensory processing

## Abstract

**Introduction:**

Identifying sensory processing problems of 12‐month‐old preterm and term children and defining their relationship with motor skills are essential for appropriate interventions and optimal sensory‐motor development. This study aimed to determine sensory processing difficulties in 12‐month‐old babies and examine their relationship with motor skills.

**Methods:**

This cross‐sectional study included 61 infants (28 preterm and 33 full‐term, ages 12 months). The infants' sensory processing skills were evaluated using the Test of Sensory Functions in Infants (TSFI), and their gross and fine motor skills were assessed with the Peabody Developmental Motor Scales‐2 (PDMS‐2).

**Results:**

Sensory processing difficulties were more common in preterm babies. Multiple linear regression models indicated a significant positive association between PDMS‐2 gross/fine motor scores and TFSI total scores, reactivity to tactile deep pressure, and ocular‐motor control in the total sample. Furthermore, there was a relationship between gross motor and adaptive motor function, and fine motor scores were found to be associated with visual‐tactile integration sensory scores.

**Conclusions:**

Preterm babies are more likely than their full‐term peers to have sensory processing problems around the age of one, which can affect their motor skills. The results support the relationship between children's sensory and motor processing skills. Practitioners such as occupational and physical therapists should be alert to this relationship in infants with sensory processing and motor problems. Taking this relationship into consideration when planning intervention programs can be a guide for an effective intervention.

## Graphical abstract

There is a relationship between children's sensory processing skills and gross/fine motor skills.

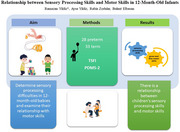

## 1 INTRODUCTION

Preterm are infants born alive before the 37 weeks of pregnancy, according to the World Health Organization (WHO) (Dbstet, 1977). Premature infants encounter more significant challenges with cognitive functions, visual‐motor and visual‐spatial abilities, language acquisition, attention, executive functions, learning, and sensory integration than infants born at full term (Kessenich, 2003). These problems arise from the detrimental effect of very premature birth on the developing brain, combined with widespread white matter abnormalities, threatening the effective processing of sensory information (Owen et al., 2013).

Sensory processing involves the capacity to receive, interpret, arrange, and react to sensory input originating from both one's own body and the external environment (Miller et al., 2012). Sensory processing disorders (SPD) are observed in premature infants due to both the immaturity of their systems and their exposure to the neonatal intensive care unit environment (NICU) (Blackburn, 1998). Sensory exposures of preterm infants in the NICU vary significantly in duration, intensity, and complexity from normal sensory stimuli in utero (Lickliter, 2000). Premature infants are exposed to loud sounds for daily physical examination, monitoring devices, intravenous catheter placement, abnormal circadian rhythm, and respiratory support. The long‐term sensory development of the infant, who has to deal with inappropriate sensory stimuli in the early period, is also negatively affected (Bartocci et al., 2001; Kramarić et al., 2017).

Children exhibiting poor sensory processing skills may face delays in developing fine and gross motor skills, alongside potential issues with balance and coordination. It has also been reported that these children have attention deficit, tactile defense, language, and visual‐spatial problems (DeGangi & Greenspan, 1989a). Among children born prematurely aged 2 to 5, unconventional sensory processing patterns have been linked with inadequate motor and cognitive development as well as executive dysfunction (Adams et al., 2015; Rahkonen et al., 2015).

The relationship between sensory processing and motor development has been examined in different age groups and diagnoses (Liu, 2013; Park, 2017). It is essential to evaluate the child's sensory processing skills at the 12th month, when the child begins to explore the environment by walking and practices trial‐and‐error experiences. Because motor development depends on active trial‐and‐error experiences, accurate sensory processing is essential during development (Hadders‐Algra, 2018). Therefore, 12‐month‐old children were included in this study. Although the relationship between motor and sensory development of preterm children in a similar age group has been examined in the literature, the relationship between fine and motor skills has not been investigated (Celik et al., 2018). This study aims to evaluate sensory processing in 12‐month‐old preterm children and investigate the association between sensory processing and gross motor and fine motor skills. In this study, we hypothesized that sensory processing skills and fine and gross motor skills are related. The second hypothesis was that preterm children had more sensory processing problems than their term peers.

## 2 METHODS

### 2.1 Participants

Approval for conducting this cross‐sectional study was granted by the Gazi University Ethics Committee (Approval Date: 05.09.2023, Approval Number: E‐77082166). Parents provided written informed consent, and the study was conducted at Gazi University, Faculty of Health Sciences, Department of Physiotherapy and Rehabilitation, from October 2023 to February 2024.

While children whose gestational age was below 34 weeks, who stayed in the NICU for at least 15 days and did not develop any complications were included in the premature group, children whose gestational age was between 37 and 41 weeks and who did not have any significant health problems were included in the term group. Children with any neurological diagnosis (such as cerebral palsy), genetic disease (such as Down Syndrome), visual or hearing impairment, or congenital heart disease were excluded from the study. G power program (version 3.1.9.2 Universit¨at Düsseldorf, Düsseldorf, Germany) was used for power analysis. In comparing the means of two independent groups, assuming an α error of .05, it was calculated that 56 babies, 28 for each group, should be included to reach 95% power. The post hoc power analysis was performed after the study, and it was found to be 85.8%, and 98.8% for the regression models with a statistical significance of α = .05, *R* ^2^‐adj = .162 and .266 (total TSFI score and total gross motor score model, total TSFI score and total fine motor score model, respectively), number of predictors = 2, and sample size = 61. All participants were born in the maternity unit of Gazi University Hospital between 2022 and 2023. The families of 78 children at 12 months old (40 preterm and 38 term) were called and invited to the study. Fourteen infants' families refused to participate in the study, and three did not meet the inclusion criteria (two of them had cerebral palsy and one of them had congenital heart disease). The study was completed with 28 preterm and 33 full‐term children who met the inclusion criteria.

### 2.2 Assessments

After parental consent was obtained, children's demographic and birth‐related information were recorded. An experienced physiotherapist performed sensory processing and motor skills in a quiet environment while the child was calm and awake. Children's sensory processing skills were evaluated with The Test of Sensory Functions in Infants (TSFI) (DeGangi & Greenspan, 1989), and their motor skills were assessed with The Peabody Developmental Motor Scales‐2 (PDMS‐2) (Folio, 1983). All assessments were performed by calculating the corrected age for preterm children.

#### 2.2.1 The Test of Sensory Functions in Infants

The TSFI is a standardized, reliable, and valid tool designed to evaluate the sensory development of infants aged 4–18 months (Jirikowic et al., 1997). Comprising 24 items, the TSFI assesses reactions across five distinct subdomains: tactile deep pressure, visual‐tactile integration, adaptive motor function, ocular motor function, and reactivity to vestibular stimulation (Jirikowic et al., 1997). Every subdomain yields an age‐normalized score, contributing to a total score calculated by summing all subdomains. The total score spans from 0 to 49, where higher values signify more typical sensory responsiveness, whereas lower scores suggest behaviors linked with sensory over‐responsivity (DeGangi & Greenspan, 1989).

#### 2.2.2 Peabody Developmental Motor Scales‐2

The PDMS‐2 assessment is used and accepted to determine a child's motor abilities. Its primary purpose is to distinguish and help identify developmental delays in children aged five and under, achieved by comparing their performance against established norms (Folio, 1983). The assessment comprises 127 gross motor and 122 fine motor items in 6 subtests and provides a fine motor assessment, gross motor assessment, and a motor quotient. Each item on the test is evaluated using a 3‐point scoring system. A score of 2 is awarded when the child effectively accomplishes the task per the predetermined criteria. A score of 1 suggests that the child's behavior displays early signs of competence, yet the conditions required for complete and successful execution still need to be fulfilled. On the contrary, a score of 0 indicates either a lack of observable signs of skill development during the attempt or the child's incapacity to attempt the task (Folio, 1983). PDMS‐2 is a scale with good test‐retest reliability in assessing motor skills.

### 2.3 Statistical analysis

The data was analyzed using IBM SPSS Statistics 25 (SPSS Inc., Chicago, IL, USA), with a significance level set at *p* < .05. Categorical data are expressed as numbers and percentages; continuous data are presented as mean ± SD. Chi‐square analysis was utilized for categorical variables to compare groups across demographic variables. Visual means (histograms and probability plots) and analytical techniques (Kolmogorov–Smirnov/Shapiro–Wilk tests) evaluated the variables' normality. In cases where parametric test assumptions were not fulfilled, the Mann–Whitney *U* test was employed to analyze differences between independent groups. Additionally, linear regression was used to explore associations between gross/fine motor skills and sensory processing scores. When the relationship was found to be significant in the simple linear regression model (*p* < .05), adjustments were made in the multiple linear regression analysis for potential confounding factors identified through descriptive analysis and literature evidence (Hediger et al., 2002). The associations between PMDS‐2 fine motor/gross motor total scores and the TSFI scores were verified within the total sample (*n*: 61) using multiple linear regression analysis with adjust for gestational age as a potential confounding factor.

## 3 RESULTS

The study encompassed 61 infants, consisting of 28 preterm and 33 full‐term infants. Detailed birth and demographic data for the children are provided in Table 1. As per the study design, the preterm group exhibited lower gestational age, birth weight, and height than the full‐term group. Parental education and other birth characteristics were similar between groups.

**TABLE 1:** Characteristics of the infants.

	Preterm group (*n* = 28)	Term group (*n* = 33)	*p*
Gestational age (week) (mean ± SD)	30.82 ± 3.07	38.45 ± 1.15	<.01^a^
Gender (F/M) *n* (%)	14/14 (50/50)	20/13 (60.6/39.4)	.41^b^
Birth weight (g) (mean ± SD)	1697.38 ± 833.02	3073.71 ± 318.26	<.01^c^
Birth height (cm) (mean ± SD)	40.92 ± 5.55	48.66 ± 2.86	<.01^c^
Type of birth (C/N) *n* (%)	17/11 (60.7/39.3)	19/14 (57.6/42.4)	.8^b^
Maternal age (year) (mean ± SD)	29.70 ± 4.84	29.93 ± 4.8	.99^c^
Mother's educational status *n* (%) Primary school High school University Master's degree	1 (3.6) 9 (32.1) 16 (57.1) 2 (7.1)	2 (6.1) 11 (33.3) 19 (57.6) 1 (3)	.93^b^
Father's educational status *n* (%) Primary school High school University Master's degree	3 (10.7) 6 (21.4) 15 (53.6) 4 (14.3)	3 (9.1) 12 (36.4) 16 (48.5) 2 (6.1)	.55^b^

SD: standard deviation; C: Cesarean section; N: normal; F: female; M: male.

^a^ Mann–Whitney *U* test

^b^ Chi‐square test.

^c^ Student's *t*‐test.

Sensory processing scores and normal/at‐risk and deficient ratios of all children are summarized in Table 2. In the TSFI total score, 28.6% of preterm children were scored as “deficient” and 21.4% were scored as “at risk.” Reactivity to tactile deep pressure, adaptive motor function, visual‐tactile integration, and TSFI total score were lower in the preterm group than in the term group (*p* < .05). Additionally, sensory processing problems were seen more frequently in the reactivity to tactile deep pressure, adaptive motor function areas, and total scores (*p* < .05).

**TABLE 2:** Comparison of infants' sensory processing skills scores.

TSFI subscales and total scores	Preterm group (*n* = 28)	Term group (*n* = 33)	*p*
	Mean ± SD *N* (%)	Mean ± SD *N* (%)	
Reactivity to tactile deep pressure Normal At risk Deficient	8.57 ± 1.66 18 (64.3) 7 (25) 3 (10.7)	9.60 ± 0.65 29 (87.9) 4 (12.1) ‐	**.003^a^** **.049^b^**
Adaptive motor function Normal At risk Deficient	12.86 ± 1.63 12 (42.9) 6 (21.4) 10 (35.7)	13.90 ± 1.01 24 (72.7) 8 (24.2) 1 (3)	**.008^a^** ** <.003^b^**
Visual‐tactile integration Normal At risk Deficient	8.25 ± 2.38 20 (71.4) 4 (14.3) 4 (14.3)	9.33 ± 0.69 29 (87.9) 4 (12.1) ‐	**.036^a^** .071^b^
Ocular‐motor control Normal At risk Deficient	1.93 ± 0.38 27 (96.4) ‐ 1 (3.6)	1.94 ± 0.35 32 (97) ‐ 1 (3)	.907^a^ .71^b^
Reactivity to vestibular stimulation Normal At risk Deficient	10.61 ± 1.75 20 (40.9) 3 (13.6) 5 (45.5)	10.67 ± 1.05 29 (87.9) 3 (9.1) 1 (3)	.462^a^ .140^b^
TSFI total Normal At risk Deficient	42.21 ± 4.77 14 (50) 6 (21.4) 8 (28.6)	45.45 ± 1.91 27 (81.8) 5 (15.2) 1(3)	**.001^a^** **.01^b^**

TSFI: Test of Sensory Functions in Infants; SD: standard deviation.

*Note*: *p* < .05 highlighted in bold.

^a^ Mann–Whitney *U* test.

^b^ Chi‐square test.

The preterm infants exhibited lower locomotion and gross motor total scores than the term group (*p* < .05). At the same time, grasping, visual motor integration, and fine motor total scores were lower in the preterm group compared to term groups (*p* < .05) (Table 3).

**TABLE 3:** Comparison of infants' gross and fine motor skills scores.

PDMS‐2 scores	Preterm group (*n* = 28)	Term group (*n* = 33)	*p*
Gross motor	93.18 ± 12.64	99.45 ± 6.77	**.009**
Stationary	35.14 ± 5.41	35.09 ± 4.14	.369
Locomotion	58.82 ± 17.99	67.15 ± 10.95	**.025**
Object manipulation	4.68 ± 2.47	5.03 ± 3.31	.814
Fine motor	97.89 ± 13.61	105.55 ± 5.96	**.013**
Grasping	36.39 ± 7.32	39.52 ± 2.45	**.047**
Visual‐motor integration	57.64 ± 13.11	63.55 ± 10.18	**.016**
Total motor	96.04 ± 14.15	102.64 ± 5.01	**.045**

*Note*: Values in bold indicate p < 0.05.

According to multiple linear regression models, both gross motor and fine motor scores of PDMS‐2 were significantly positively correlated with TFSI total scores, tactile deep pressure response, and ocular‐motor control. In addition, while gross motor and adaptive motor function were related, fine motor scores were related to visual‐tactile integration sensory scores (Table 4).

**TABLE 4:** Multiple linear regression model in the full sample to evaluate the relationship between PDMS‐2 (gross and fine motor) scores and TSFI scores.

Outcome	TSFI scores	*β*	SE	*p* Value
PDMS‐2 gross motor	Constant	50.49	11.38	
	Total	0.54	0.15	**<.001**
	Gestational age	0.17	0.29	.12
	Constant	64.06	11.85	
	Reactivity to tactile deep pressure	0.38	0.71	**.003**
	Gestational age	0.13	0.33	.30
	Constant	54.46	11.11	
	Adaptive motor function	0.53	0.37	**<.001**
	Gestational age	0.20	0.29	.073
	Constant	69.68	12.19	
	Visual‐tactile integration	0.25	0.78	.06
	Gestational age	0.14	0.35	.28
	Constant	53.02	14.02	
	Ocular‐motor control	0.34	3.95	**.007**
	Gestational age	0.23	0.33	.065
	Constant	64.25	13.24	
	Reactivity to vestibular stimulation	0.66	0.24	.06
	Gestational age	0.34	0.22	.08
PDMS‐2 fine motor	Constant	56.59	11.29	
	Total	0.73	0.14	**<.001**
	Gestational age	−0.04	0.29	.69
	Constant	92.30	14.34	
	Reactivity to tactile deep pressure	0.29	0.95	**.036**
	Gestational age	−0.09	0.44	.51
	Constant	74.61	18.29	
	Adaptive motor function	0.25	0.99	.055
	Gestational age	0.02	0.40	.98
	Constant	75.78	11.09	
	Visual‐tactile integration	0.55	0.69	**<.001**
	Gestational age	−0.08	0.31	.40
	Constant	48.57	12.25	
	Ocular‐motor control	0.71	2.97	**<.001**
	Gestational age	0.07	0.23	.44
	Constant	82.03	17.20	
	Reactivity to vestibular stimulation	0.21	0.78	.12
	Gestational age	0.07	0.42	.60

TSFI: Test of Sensory Functions in Infants; β: standardized coefficient; SE: standard error.

*Note*: The constant represents the anticipated mean value of the PMDS‐2 score under the condition that the TSFI scores are set to zero.Values ​​in bold indicate p 〈 0.05.

## 4 DISCUSSION

This study aimed to assess sensory processing abilities in preterm infants and explore the relationships between gross and fine motor skills and sensory processing skills in both full‐term and preterm infants. In the study, it was found that preterm infants had more sensory processing difficulties at the age of 12 months than their term peers. Moreover, a correlation was observed between sensory processing and gross and fine motor skills among preterm and full‐term infants.

Children with low gestational age are more prone to atypical sensory processing (Chorna et al., 2014b; Rahkonen et al., 2015), and sensory processing issues are more prevalent in preterm children than in those born at full term (Cabral et al., 2015; Celik et al., 2018). Case‐Smith et al. (1998) reported that general and tactile sensory processing problems are more common in preterm infants with a gestational age of less than 36 weeks. Wiener et al. (1996) found that 10‐ to 12‐month preterm infants with a gestational age of less than 32 weeks had lower tactile, vestibular‐proprioceptive and total sensory processing scores. Chorna et al. (2014a) found that tactile and vestibular‐proprioceptive processing were most affected in preterm infants with a gestational age of less than 30 weeks and a birth weight of less than 1500 g. Cabral et al. (2015) found a significant difference in 4‐ to 6‐month TSFI total and tactile processing scores in infants with a gestational age of less than 37 weeks, who stayed in the NICU for at least 1 day. Celik et al. (2018) found that preterm infants scored lower in the areas of response to tactile deep pressure, visual–tactile integration, and response to vestibular stimuli. These sensory processing problems in preterm children are reported to be related to exposure to harmful sensory stimuli during the early stages of development in the NICU (Als et al., 2004; El‐Metwally & Medina, 2020; Nevalainen et al., 2008). In the present study, similar to the literature, there were differences between the groups in the areas of reactivity to tactile deep pressure, adaptive motor function, visual‐tactile integration. There was no difference between the groups in terms of ocular‐motor control and reactivity to vestibular stimulation. Compared to other sensory systems, the development of the visual system is still very primitive when the baby is born. Because the visual system cannot receive sufficient stimulation in the dark environment of the uterus. The development of visual perception, recognition, discrimination and visual memory begins immediately after birth and continues until the age of 5–6 (Graven & Browne, 2008). The lack of significant differences in oculomotor control in the present study suggests that by the time they reach 12 months of corrected age, preterm infants may have reached a developmental level comparable to that of term infants in this area. This finding is consistent with some studies showing that although preterm infants may experience delays in certain areas of development, they can catch up in sensory and motor development, especially when appropriate early interventions are provided (Apaydın et al., 2023; Oh & Heo, 2024). It is possible that oculomotor control, which involves the coordination of eye movements for visual pursuit and fixation, matures at a similar rate regardless of gestational age at birth, provided that infants have access to a supportive environment postnatally. Future studies could investigate whether specific factors, such as the level of neonatal care or post‐discharge support, contribute to this apparent normalization. Similarly, the lack of differences in response to vestibular stimulation between the two groups suggests that the ability to respond to changes in head position and movement, which are crucial for balance and spatial orientation, may develop in the same way in preterm and term infants by 12 months of age. This result may indicate that the vestibular system, which is responsible for sensing motion and gravity, is flexible and can develop adequately despite premature birth. Given the critical role of vestibular function in general motor development, this finding is encouraging and highlights the potential for plasticity in sensory systems among preterm infants. However, although no differences were detected at this particular time point, it is important to consider that longitudinal studies may reveal differences in the development of vestibular‐related functions such as balance and coordination as children grow.

Sensory experiences provide crucial input for motor skill acquisition, as sensory feedback guides the refinement of movements and coordination. Sensory processing disorders are associated with movement disorders such as stereotyped movements (Gal et al., 2010). Eeles et al. (2013) demonstrated that sensory processing abilities of very preterm children were related to language, cognitive, and motor development at 2 years of age.

Cabral et al. (2015) identified a correlation between motor development and sensory processing in infants aged 4 to 6 months. Celik et al. (2018) showed a relationship between motor development and sensory processing in 10‐ to 12‐month‐old infants. de Paula Machado et al. (2019) reported in their study that sensory development in 12‐month‐old premature infants is related to motor development. Kara et al. (2020) examined the relationship between neuromotor development and sensory development in premature babies in their studies. It was stated in their studies that sensory development and motor development of premature babies in the 4th month were strongly related (Kara et al., 2020). In this study, a relationship was found between gross motor skills and sensory processing skills, parallel to the literature.

Studies investigating sensory processing skills in the literature have generally examined the relationship between gross motor development and have not focused on fine motor development (Cabral et al., 2016; Celik et al., 2018). Unlike the studies in the literature, the relationship between fine motor skills and sensory processing skills was also examined in the current study. It was found that fine motor skills and sensory processing skills were related, especially between visual‐tactile integration and ocular motor control. Vision, oculomotor control, and sensory input are critical in fine dexterity (Niechwiej‐Szwedo et al., 2017). According to Chorna et al. (2014b), deficient ocular‐motor control was associated with poorer motor scores in early childhood. de Paula Machado et al. (2019) identified a significant association between Bayley 3 motor scores and the ocular motor control domain in infants at 12 months of age. The mechanisms behind motor control and eye movement control involve neuroanatomically standard networks (Diamond, 2000). The same networks that govern general motor movements overlap with oculomotor networks, and vestibular‐ocular connections effectively develop postural control and adaptive motor responses (Chorna et al., 2014b). These can explain the relationship between fine motor/gross motor and oculomotor control.

Motor and sensory developments are intertwined during infancy and early childhood. Sensory feedback guides improving movements and coordination; sensory experiences provide essential input for motor skill acquisition. Visual input helps infants coordinate movements to reach objects, while tactile feedback helps them grasp and manipulate objects. Sensory‐motor experiences play a role in developing functions such as reaching and grasping (Corbetta & Snapp‐Childs, 2009). The tactile feedback provided by mechanoreceptors in the skin and joints plays a crucial role in dynamically adjusting gross motor functions like walking and fine motor functions like grasping throughout early development and lifespan (Metcalfe et al., 2005; Soechting & Flanders, 2008). This may explain the relationship between fine motor skills, visual‐tactile integration, and reactivity to tactile deep‐pressure domains.

A limitation of the study is that preterm infants were not classified by gestational age (e.g., very preterm, extremely preterm). This could have potentially masked the association between younger gestational age and motor deficiencies. Therefore, it is recommended that future studies examine these associations in specific gestational age groups. This study also focused on a single developmental point. Comprehensive studies that focus on all areas of development, not just sensory and motor skills, are needed. The another limitation of this study was that the relationship between sensory and motor skills was evaluated in only 12 months. Longitudinal studies are needed to examine the effects of sensory difficulties in preschool and school‐age periods and to understand their impact on other developmental areas, such as motor‐cognitive language skills. The importance of sensory and motor evaluations in the follow‐up of preterm babies at risk for sensory processing problems should be emphasized for health professionals such as physiotherapists, occupational therapists, and parents.

## 5 CONCLUSION

Research in the literature emphasizes sensory processing and motor development, underlining the relationship between these developmental domains (Cabral et al., 2015; Celik et al., 2018; de Paula Machado et al., 2019). This study focused on the relationship of sensory processing with gross and fine motor skills. Our results support the relationship of both gross motor and fine motor skills with sensory processing. Both processes rely on and support each other throughout infancy, childhood, and beyond, providing the basis for adaptive functioning and overall development. Understanding the relationship between sensory and motor development can be helpful for both the evaluation and therapy process. Designing effective interventions that include motor and sensory approaches together, especially for infants with motor and sensory problems, is important to support optimal development.

## AUTHOR CONTRIBUTIONS

**Ramazan Yildiz**: Conceptualization; investigation; methodology; formal analysis; writing—review and editing; writing—original draft. **Ayse Yildiz**: Investigation; writing—review and editing; methodology; writing—original draft. **Rabia Zorlular**: Methodology; investigation. **Bulent Elbasan**: Conceptualization; writing—review and editing; methodology; supervision.

## FUNDING INFORMATION

This research did not receive any specific grant from funding agenciesin the public, commercial, or not‐for‐profit sectors.

### PEER REVIEW

The peer review history for this article is available at https://publons.com/publon/10.1002/brb3.70052.

## Data Availability

The data that support the findings of this study are available from the corresponding author upon reasonable request.
